# Comparison of gadolinium dose and acquisition time for late gadolinium enhancement at 3.0 T

**DOI:** 10.1186/1532-429X-16-S1-O94

**Published:** 2014-01-16

**Authors:** Adelina Doltra, Alex Skorin, Bernhard Schnackenburg, Christoph Klein, Eckart Fleck, Sebastian Kelle

**Affiliations:** 1 Cardiology, German Heart Institute Berlin, Berlin, Germany; 2 Philips Healthcare Systems, Hamburg, Germany

## Background

Although late gadolinium enhancement (LGE) is a widely used technique in daily clinical practice, the optimal contrast dose and time of acquisition at 3.0 T is unknown. Aim of our study was to compare different contrast doses and acquisition times for LGE imaging at 3.0 T.

## Methods

34 patients with chronic myocardial infarction were randomized to 0.1 (n = 12), 0.15 (n = 11) and 0.2 (n = 11) mmol/Kg of gadolinium contrast (gadobenate dimeglumine - MultiHance^®^). T1-weighted inversion recovery gradient echo sequences were performed at 5, 10, 15 and 20 minutes post-administration of contrast in all groups, with an individually adapted trigger delay at every single time point. Signal-to-noise ratio (SNR) of the scar, contrast-to-noise ratio (CNR) of the scar in comparison to healthy myocardium and the percentage of enhanced area volume relative to the global myocardium were quantified. A 4-point score was used to assess image quality in all studies.

## Results

**Figure 1:**
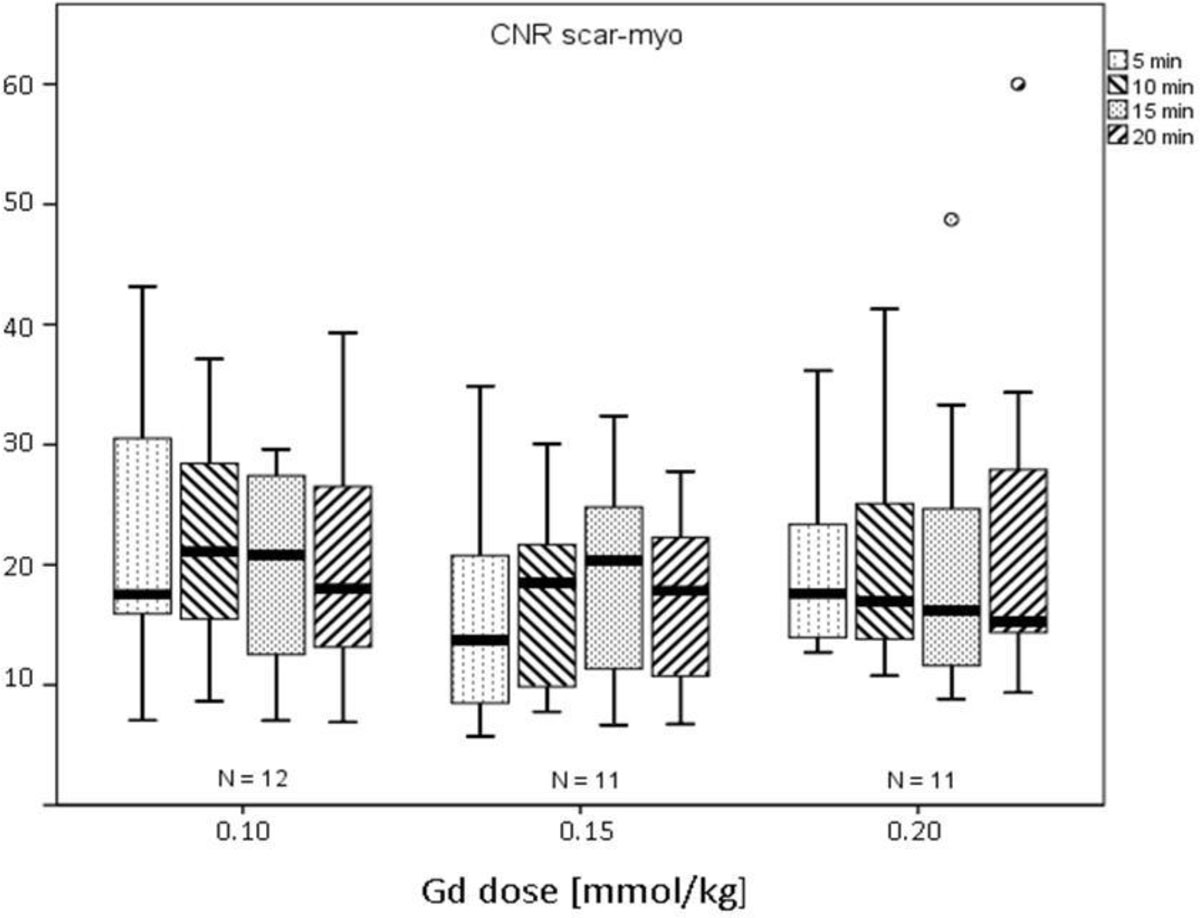
**Contrast-to-noise ratio of the scar tissue compared to the non-enhanced myocardium at 3 doses and 4 different time-points**.

**Figure 2:**
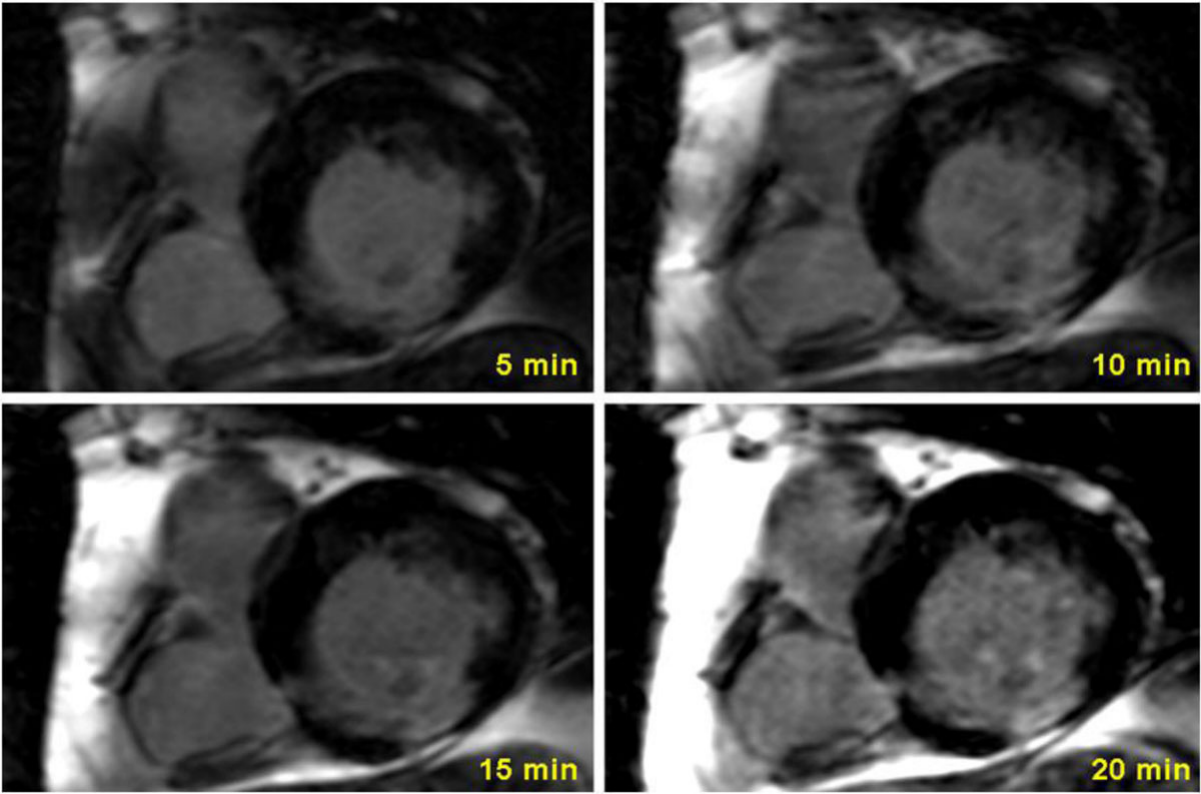
**Short axis views of a patient with an infarction of the basal inferior wall acquired 5, 10, 15 and 20 min after the administration of 0.10 mmol/kg Gd**.

No differences were observed in SNR and CNR (see Figure 1), neither between the doses being evaluated nor between the different acquisition times. Regarding enhanced area volume, at 0.1 mmol/kg of Gd contrast, imaging at 5 min yielded lower enhanced area volumes in comparison to 15 and 20 minutes (7.5 ± 4.3 vs 9 ± 3.9 vs 9.5 ± 5.4, p = 0.03 and p = 0.02, respectively) (Figure 2). No significant differences between imaging times were observed at 0.15 and 0.2 mmol/kg. Finally, when analyzing image quality at 0.20 mmol/kg Gd significant changes were observed between 5 and 15 min (2.6 ± 0.5 vs 3.2 ± 0.8, p = 0.014), 5 and 20 min (2.6 ± 0.5 vs 3, 2.6 ± 0.5 vs 0.8, p = 0.046) as well as 10 and 15 min (2.7 ± 0.5 vs 3.2 ± 0.8, p = 0.025), with no significant differences in the remaining imaging times and doses.

## Conclusions

In LGE imaging at 3.0T low doses of gadolinium and early acquisitions perform equally well in terms of SNR and CNR, although a trend towards poorer image quality with early acquisitions is noted. When using lower contrast doses, early acquisition is associated with lower enhanced area volumes. As a consequence, late acquisition is preferable. Studies with sufficient diagnostic quality could be obtained using shorter protocols with low contrast doses.

## Funding

None.

